# Variant Guillain-Barré Syndrome in a Patient with Non-Hodgkin's Lymphoma

**DOI:** 10.1155/2015/979237

**Published:** 2015-08-11

**Authors:** R. H. Bishay, J. Paton, V. Abraham

**Affiliations:** ^1^St George Hospital, Kogarah, Sydney, NSW 2217, Australia; ^2^Shoalhaven District Memorial Hospital, Nowra, NSW 2541, Australia; ^3^Sydney Medical School, University of Sydney, Sydney, NSW 2006, Australia

## Abstract

We report a 72-year-old female patient with diffuse large B cell non-Hodgkin's lymphoma (NHL) with previous treatment with standard chemotherapy presenting as an acute, ascending, sensorimotor polyneuropathy. Nerve conduction studies and lumbar puncture supported a rare, but ominous, axonal variant of Guillain-Barré Syndrome (GBS) known as acute motor and sensory axonal neuropathy (AMSAN), which is distinguished from the more common, acute demyelinating forms of GBS. Previous reports have largely focused on toxicities secondary to chemo- or radiotherapy as a major contributor to the development of acute neuropathies in malignancy. Clinicians should also be mindful of direct neoplastic invasion or, less commonly, paraneoplastic phenomenon, as alternative mechanisms, the latter possibly reflecting immune dysregulation in particularly aggressive lymphomas. At the time of writing, this is the first report in the literature of an axonal variant of GBS in a patient with diffuse large B cell NHL. A discussion regarding common and uncommon neuropathies in haematological malignancies is made, with a brief review of the anecdotal evidence supporting a paraneoplastic association with GBS or its variant forms in the setting of lymphoma.

## 1. Introduction

It is now recognised that aggressive haematological malignancies are associated with a variety of both central (CNS) and peripheral nervous system (PNS) pathologies. A growing body of literature linking haematological malignancies such as non-Hodgkin's lymphoma (NHL) with acute inflammatory demyelinating disease such as Guillain-Barré Syndrome (GBS) has been previously reported [1–3]. Although rare, GBS or its variant forms should be suspected in any patient with an aggressive form of NHL with progressive, neurological symptoms.

Non-Hodgkin's Lymphoma is a neoplastic transformation of immune cells that are primarily located within lymphoid tissues, which are usually of B cell or T cell origin. Its incidence rises with age and is slightly more common in men. It is known that NHL can affect both the CNS and the PNS, the latter in approximately 5% of cases [1]. A variety of modes of neurological disease in patients with NHL have been reported, which include direct invasion of subarachnoid tissue (i.e., neurolymphomatosis), spinal cord compression, plexopathies, and leptomeningeal infiltration or, less commonly, spread to cerebral parenchyma, as paraneoplastic phenomenon, side-effects of chemotherapy (especially vinca alkyloids, cytarabine or intrathecal methotrexate), radiotherapy (i.e., radiation myelopathy), or due to opportunistic infections (commonly herpes zoster) [1, 2]. Patients with neurological symptoms due to NHL may commonly present headaches, seizures, lethargy, focal neurological symptoms, or paralysis. Less commonly, it may present with spinal cord compression (pain, weakness, sensory disturbance, urinary and bowel incontinence, and areflexia) or meningitis [2].

Acute inflammatory demyelinating polyradiculoneuropathy (AIDP), also referred to as GBS, is the most common form of acute flaccid paralysis worldwide and is a neurological emergency. The diagnosis of GBS is clinical and supported by the classical findings of symmetrical, ascending lower limb weakness and areflexia. Supportive investigations include nerve conduction studies (NCS) which classically show prolonged distal latencies (indicating distal conduction block), reduced conduction velocities, and prolonged or absent F waves (reflecting proximal nerve and nerve root disease) [4]. Other supportive diagnostic features include the albuminocytologic dissociation in cerebrospinal fluid (CSF, protein > 0.55 g/L without elevation in white blood cell count) and exclusion of competing differential diagnoses. The development of symptoms is typically less than 4 weeks and if longer than 8 weeks is usually classified as chronic inflammatory demyelinating polyradiculoneuropathy (CIDP).

The pathophysiological basis for both AIDP and CIDP is not fully known but is thought to be associated in two-thirds of cases with an antecedent upper respiratory or gastrointestinal tract infection, most commonly* Campylobacter jejuni*,* Cytomegalovirus*, or Epstein-Barr virus. Treatment outcomes with either intravenous immunoglobulin (IVIG) or plasma exchange are similar, with no role for the use of corticosteroids. Long-term prognosis leaves 20% of patients with a long-term disability and carries 5% mortality [5].

Variant forms of GBS are now recognised and should alert the clinician to the many varieties of presentation. The commonest presentation is the classical form of GBS or AIDP and presents in 85–90% of cases. The pathophysiology is primarily due to demyelination of Schwann cell surface membranes due to autoimmunity from molecular mimicry of surface epitopes. Other demyelinating forms include Miller-Fisher Syndrome (MFS), Bickerstaff encephalitis, and pharyngeal-cervical-brachial disease; these three diseases have been suggested to form a cluster of disorders known as anti-GQ1b antibody syndrome given the high prevalence of the antibody (>96%) [5].

In contrast to demyelination, autoimmunity against axonal epitopes gives rise to axonal GBS such as acute motor axonal neuropathy (AMAN) and acute motor and sensory axonal neuropathy (AMSAN). As the name suggests, AMAN involves motor nerves with early and severe respiratory involvement; it often affects children and young adults and 75% of cases have positive serology for* Campylobacter jejuni* and are associated with other antibodies (anti-GM1 and anti-GD1a). In contrast, AMSAN includes both sensory (dorsal) and motor (ventral) roots, is more severe with respiratory and bulbar involvement, as reported in our index patient, and carries a poorer prognosis. In contrast to classical GBS, diagnosis with NCS shows small or absent motor (i.e., AMAN) or sensory and motor amplitudes (i.e., AMSAN), with normal distal latencies and absence of conduction block [4].

## 2. Case Presentation

In November 2014, a 72-year-old female presented to the emergency department of a rural hospital in New South Wales with a mechanical fall in the context of a one-week history of generalized weakness, dizziness, poor oral intake, and vomiting as well as constipation. Her past medical history included stage IV diffuse large B cell non-Hodgkin's lymphoma diagnosed 1-year earlier, multinodular goitre, Bell's palsy, gout, and knee osteoarthritis. Her current medications included allopurinol 100 mg daily, paracetamol 1 g QID, and celecoxib 200 mg daily. The patient was diagnosed with NHL when she presented with “B symptoms” (i.e., fever, weight loss, and drenching night sweats) but with no symptoms of nodal enlargement of the neck, abdomen, or other neurological symptoms to suggest CNS disease. Initial time-of-flight (ToF) fluorodeoxyglucose (FDG) with integrated positron emission tomography (PET) and computed tomography (CT) revealed tracer uptake at the left olfactory recess of the nasal cavity, left pterygopalatine fossa, left masticator space, left sacral ala, and left thyroid nodule as well as ascending colon. She received 8 cycles of R-CHOP-21 chemotherapy (i.e., rituximab, cyclophosphamide, doxorubicin, vincristine, and prednisone) and intrathecal methotrexate (12.5 mg with each cycle) given the nasal involvement. She received consolidation involved-field radiotherapy (45 Gy) 1 month following completion of chemotherapy. A restaging PET/CT 12 weeks following treatment revealed increased tracer uptake in the left olfactory recess of the nasal cavity medially to the left ethmoid sinus, abutting the ethmoid bone, which was suspected of disease recurrence but with stable appearances elsewhere (Deauville score = 4).

The patient's management included further chemotherapy but presented to a rural hospital with dehydration and lethargy. She was found to be hypertensive (170/80 mmHg) with no postural drop. She was haemodynamically stable, with a heart rate of 90 beats/minute, respiratory rate of 20/min, oxygen saturations of 97% on room air, and afebrile (36.5°C). Her cardiorespiratory examination was normal. Abdominal examination failed to reveal organomegaly or palpable lymph nodes. Per rectal examination revealed hard stool. Neurologically, there was decreased power bilaterally in the upper and lower limbs, slightly worse on the left with decreased shoulder abduction (left 3/5, right 4/5), hip flexion (left and right 3/5), and dorsiflexion (left 0/5, right 3/5). There was areflexia of the upper (brachioradialis) and lower limb (ankle and knee jerk) responses bilaterally. Clonus was absent and plantar response was equivocal. Cranial nerve (CN) deficits were seen to involve the right facial nerve (CN VII) with weakness of the right side and hypoglossal nerve (CN XII) with tongue deviation to the left. There was also moderate pharyngeal dysphagia and mild dysarthria. Anal tone was diminished.

The patient was kept nil by mouth and a nasogastric tube was inserted for feeding. Electrolytes, renal function, full-blood count, and thyroid function were normal. Initial spirometry revealed mild obstruction (FEV1 67%, FEV1/FVC 0.69). Computed tomography (CT) of the brain, neck, chest, and abdomen revealed a solid lesion of the left parotid gland, with complete opacification of both maxillary antra as well as peripherally calcified lymph nodes throughout the abdomen, presumed to be due to previously treated lymphoma. There was no demonstrated mediastinal or hilar lymphadenopathy. Magnetic resonance imaging (MRI) of the brain and spine revealed only white matter chronic ischaemic disease, with normal cerebral hemispheres, cerebellum, and brainstem and no abnormal parenchymal enhancement. There was confirmatory evidence of complete opacification of the maxillary antra without evidence of focal bony destruction. Interestingly, it was noted that there is enhancement of the dorsal and ventral nerve roots of the cauda equina.

A provisional diagnosis of an acute demyelinating polyradiculoneuropathy or GBS was made and the patient was immediately transferred to a major tertiary referral hospital. Lumbar puncture revealed a clear fluid with elevated protein (1.35 g/L), marginally elevated white cell count (9 × 10^6^/L), and normal glucose (2.9 mmol/L). There was no growth on culture or polymerase chain reaction (PCR). Cytology reported scattered atypical lymphocytes and monocytes and, unfortunately, there was no sufficient sample for immunophenotyping. Nerve conduction studies revealed absent sensory and motor responses as well as decreased amplitude in the upper and lower limbs, absent H reflexes, and reduced F waves in the upper and lower limbs. The results supported a diagnosis of acute motor and sensory axonal neuropathy (AMSAN), a variant form of Guillain-Barré Syndrome. Faecal culture for* Campylobacter jejuni* as well as anti-GQ1B antibody was negative. The patient was treated with two 5-day courses of IVIG (0.4 g/kg/day) and underwent a cycle of chemotherapy (R-CHOP) and intrathecal methotrexate. Unfortunately, despite a trial of treatment, the patient deteriorated clinically with decreasing cognitive state, haemodynamic instability from nonneutropenic sepsis, and multiorgan failure, including heart failure with acute pulmonary oedema, acute kidney injury, and coagulopathy. She required intubation and mechanical ventilation with inotropic support, broad spectrum intravenous antibiotics, diuretic therapy, and correction of her coagulopathy with fresh frozen plasma and vitamin K. After a lengthy discussion with the next of kin and considering her poor prognosis, the patient was palliated and died two weeks later.

## 3. Discussion

The rare association between the development of GBS and NHL is being increasingly recognised in the literature and is thought to occur in less than 0.3% of NHL patients [1, 6, 7]. Previous reports have documented the development of GBS during or shortly following R-CHOP chemotherapy for NHL [1, 7–9] or in CNS Burkitt's lymphoma [10]. There are at least two case reports of variant GBS in association with NHL: one report of AMSAN, as in our index patient, in the setting of currently treated Burkitt's lymphoma [6], with another citing the presence of Miller-Fisher Syndrome in the context of diffuse large B cell NHL [11].

In contrast to previous studies where clinical outcomes have been favourable following a trial of IVIG and standard chemotherapy, unfortunately our patient deteriorated and died shortly following treatment. This is perhaps due to the degree of immune dysregulation, the burden of disease in the patient considering facial and sinus invasion, older age, and the ominous prognosis of AMSAN [12]. Furthermore, previous studies have observed poorer prognosis in patients with both solid and haematological malignancies and the coexistence of GBS [13]; interestingly, the same study reported that malignancy in their population cohort was more than twice as likely to occur in patients with GBS though this observation was limited by bias.

Exactly why ascending polyneuropathies such as GBS and its variants occur in the setting of haematological or solid organ malignancy has been a matter of controversy. A number of proposed mechanisms have been made and some authors maintain that the association is merely coincidental, possibly as a result of immune dysregulation secondary to rituximab use, autoimmunity due to oncoantigen inducing misdirected immune targets against epitopes present in the PNS, or immunosuppression with or without opportunistic infections [1, 2, 8, 13, 14]. The latter is suggested as previous cases have reported the incidence of GBS in immunocompromised (e.g., HIV) and immunosuppressed patients following organ transplantation [15].

There is speculation as to whether GBS in haematological malignancies represents a true paraneoplastic process or is rather more reflective of immune dysregulation, especially given the elevated levels of interleukin-6 in the CSF of patients with GBS and CIDP [6, 14, 16]. Strictly speaking, GBS and its variants are not classified as paraneoplastic phenomenon and previous efforts to define paraneoplastic diseases have been made [17–19]. They include the presence of a characteristic syndrome associated with a particular cancer, remission of neurological symptoms after successful treatment of the malignancy, presence of antibodies to antigens expressed by tumour and nervous system cells, and a short latency between the onset of neuropathy and malignancy. Clearly, not all these requirements were met in our patient. Nonetheless, paraneoplastic phenomenon has been reported to occur in 10–20% of all malignancies and has a predilection for aggressive solid organ tumours (e.g., small cell lung carcinoma) and lymphomas (e.g., Hodgkin's Lymphoma, diffuse large B cell NHL, or T cell NHL) [1, 7, 13]. We did not investigate particular paraneoplastic antibodies or CSF interleukin levels in our patient and, despite treatment with chemotherapy, the patient's clinical status deteriorated. Future studies to ascertain particular oncogenic, antinerve tissue epitopes resulting in a GBS phenotype in animal models are lacking and would solidify GBS and its variant forms as a paraneoplastic disease entity.
